# An Enhanced Hybrid Screening Approach to Identify Potent Inhibitors for the SARS-CoV-2 Main Protease From the NCI Compound Library

**DOI:** 10.3389/fchem.2022.816576

**Published:** 2022-02-17

**Authors:** Shuhua G. Li, Kai S. Yang, Lauren R. Blankenship, Chia-Chuan D. Cho, Shiqing Xu, Hongbin Wang, Wenshe Ray Liu

**Affiliations:** ^1^ Texas A&M Drug Discovery Laboratory, Department of Chemistry, Texas A&M University, College Station, TX, United States; ^2^ Center for Biomedical Informatics, College of Medicine, Texas A&M University, Houston, TX, United States; ^3^ Institute of Biosciences and Technology and Department of Translational Medical Sciences, College of Medicine, Texas A&M University, Houston, TX, United States; ^4^ Department of Biochemistry and Biophysics, Texas A&M University, College Station, TX, United States; ^5^ Department of Molecular and Cellular Medicine, College of Medicine, Texas A&M University, College Station, TX, United States

**Keywords:** COVID-19, SAR-CoV-2, main protease, virtual screening, statistical analysis

## Abstract

The emergence and rapid spread of SARS-CoV-2, the pathogen of COVID-19, have caused a worldwide public health crisis. The SARS-CoV-2 main protease (Mpro) is an essential enzyme for the virus and therefore an appealing target for the development of antivirals to treat COVID-19 patients. Recently, many *in silico* screenings have been performed against the main protease to discover novel hits. However, the actual hit rate of virtual screening is often low, and most of the predicted compounds are false positive hits. In this study, we developed a refined virtual screening strategy that incorporated molecular docking and post-docking filtering based on parameters including molecular weight and surface area, aiming to achieve predictions with fewer false positive hits. We applied this strategy to the NCI library containing 284,176 compounds against Mpro. *In vitro* potency analyses validated several potent inhibitors and thus confirmed the feasibility of our virtual screening strategy. Overall, The study resulted in several potent hit Mpro inhibitors, in which two inhibitors have IC_50_ values below 1 μM, that are worth being further optimized and explored. Meanwhile, the refined virtual screen strategy is also applicable to improve general *in silico* screening hit rates and is useful to accelerate drug discovery for treating COVID-19 and other viral infections.

**Graphical Abstract:**
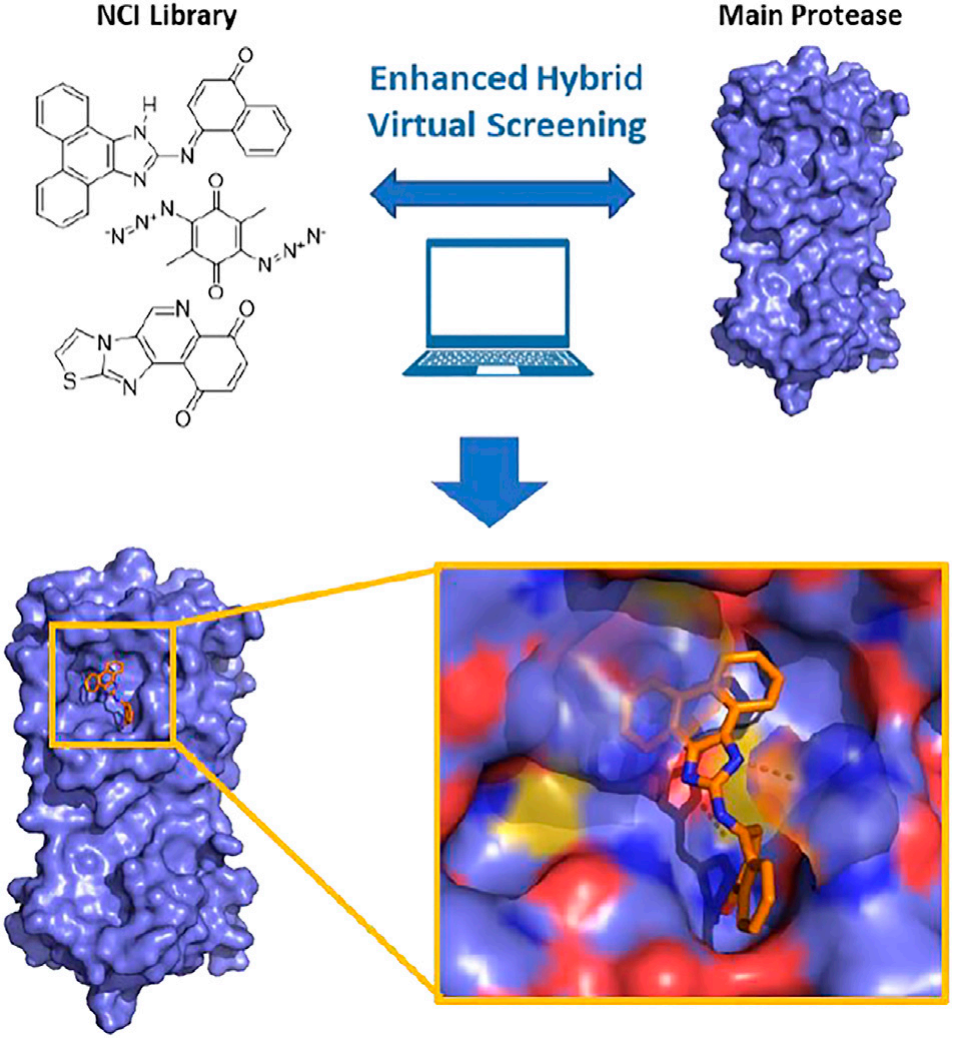


## Introduction

In the past 2 decades, coronaviruses (CoV) have caused three major worldwide infectious disease outbreaks including the severe acute respiratory syndrome (SARS) in 2003 (Lee et al., 2003; Cheng et al., 2007), the Middle East respiratory syndrome (MERS) in 2012 (Zaki et al., 2012; de Groot et al., 2013) and the coronavirus disease 2019 (COVID-19) (Gates, 2020). Their CoV pathogens are namely SARS-CoV, MERS-CoV and SARS-CoV-2, respectively. Compared to the previous two CoV outbreaks, COVID-19 has a worldwide impact that has been so severe that it is often compared to the 1918 influenza pandemic (Gates, 2020; Morens et al., 2020). According to the statistics that was released from the World Health Organization (WHO) on 12 Jan 2021, the confirmed worldwide COVID-19 cases have exceeded 312 million, of which more than 5 million patients have succumbed to death (WHO (2022) COVID-19 Dashboard). The typical COVID-19 symptoms include shortness of breath, cough and fever. In advanced cases, the infection could lead to dyspnea, pneumonia, kidney failure and even death (Huang et al., 2020). Institutions and companies around the world have been exerting much effort in rapidly developing vaccines and drugs to fight COVID-19. Three COVID-19 vaccines developed by Pfizer/BioNTech, Moderna and Johnson and Johnson have been approved or authorized by U.S. Food and Drug Administration (FDA) for human immunization in the United States. Although vaccines are promising in containing the pandemic, their availability does not diminish the urgent need for other effective antiviral drugs. Existing COVID-19 vaccines target the membrane Spike protein of SARS-CoV-2, which is highly mutable (Morse et al., 2020). New viral strains with critical mutations in Spike have emerged in various countries such as the United Kingdom, South Africa and Indian (Vilar and Isom, 2021). The efficacy of vaccines against these strains (as well as newer yet-to-emerge ones) is uncertain. Vaccines are also preventative, making them not an option for the treatment of COVID-19 patients. Hence, in addition to vaccines, it is necessary to develop therapeutic drugs for both prevention and treatment as we are now observing new waves of the COVID-19 pandemic from Delta and Omicron strains. As a quick access to effective antivirals, drug repurposing has been broadly conducted (Vatansever et al., 2021a). Although the FDA has approved some repurposed drugs including remdesivir to treatment COVID-19, most current evidence have shown that these repurposed drugs provide mild benefits to patients (Wang et al., 2020). In the context of the disastrous damage of COVID-19 to public health, civil society and the global economy, the search for effective drugs against SARS-CoV-2 is in urgent demand.

SARS-CoV-2 is a positive RNA virus that belongs to the *betacoronavirus* genus of the *coronaviridae* family under the order *Nidovirales* (Helmy et al., 2020). The genome of SARS-CoV-2 is composed of 14 open reading frames that encode 4 structural proteins, 16 nonstructural proteins (Nsps) and several accessory proteins (Astuti and Ysrafil, 2020). SARS-CoV-2’s entry into a host cell is initiated by binding the viral Spike protein to the cellular receptor angiotensin-converting enzyme 2. Viral fusion to the host cell endosome is promoted by the cellular surface serine protease. After entry and release of viral genomic RNA, two large open reading frames ORF1a and ORF1ab are translated, producing viral polyproteins pp1a and pp1ab. Both pp1a and pp1ab need to undergo proteolytic cleavage to form 16 Nsps that are essential for the virus in its reproduction and pathogenesis. The proteolytic cleavage of pp1a and pp1ab is an autocatalytic process. Two internal polypeptide regions, Nsp3 and Nsp5, possess cysteine protease activities that cleave themselves and all other Nsps from the two polypeptides. Nsp3 is commonly referred to as papain-like protease (PLpro) and Nsp5 as 3C-like protease (3CLpro) or, more recently, main protease (Mpro) (V'Kovski et al., 2021). Although we have yet to fully understand the SARS-CoV-2 biology and COVID-19 pathogenesis, current research results have established that activities of both PLpro and Mpro are essential for the viral replication and pathogenesis. Of the two proteases, Mpro processes 13 out of the total 16 Nsps. Therefore, small-molecule medicines that can potently inhibit SARS-CoV-2 Mpro are potentially effective treatment options for COVID-19 (Yang et al., 2021).

Recently, *in silico* screening has been used to identify potential drugs against SARS-CoV-2 and results from these investigations have been reported (Rakib et al., 2020; Mahmud et al., 2021a; Mahmud et al., 2021b; Jang et al., 2021; Khan et al., 2021; Rakib et al., 2021). Natural product studies have been done recently using computational approaches to study different inhibitors for SARS-CoV-2 Mpro enzyme (Rakib et al., 2020; Khan et al., 2021). The results of the computational approaches can then be verified further by using *in vitro* and *in vivo* experiments on the compounds identified with the highest potential binding affinities to SARS-CoV-2 Mpro. This approach was recently used to identify ethaselen, a selenium containing heterocycle, that is on its way to becoming a potential drug to combat SARS-CoV-2 infections (Rakib et al., 2021). This combined approach can be expanded past the current pandemic into other infections, such as was recently done to investigate drug candidates against MERS-CoV by targeting S1-NTD (Bouback et al., 2021). Computational biological techniques allow for increased efficiency and lowers the total compounds that need to be further tested to those most likely to succeed.

However, the actual hit rate of most virtual screening is low. Many predicted drug candidates are false positives (Vatansever et al., 2021b). This is partially due to the difficulties in accurately modeling and predicting protein-ligand binding free energy. This body of research suggests that it is difficult to identify false positives based on docking and simulation results alone. Therefore, better strategies are required to increase the hit rates. In this work, we incorporated effective filtering methods after molecular docking to improve the hit rate. Rigid and flexible docking were conducted parallelly. The raw docking results were then submitted to an analysis based on their molecular weights and surface area. Compounds that deviated far from their average binding score levels were identified as candidates and retained for further individual inspection. This virtual drug screening strategy, comprising the rigid/flexible docking, post-docking filtering and individual inspection, was applied to identify hits candidates targeting SARS-CoV-2 Mpro, using a recently determined crystal structure (Jin et al., 2020). A collection of 284,176 NCI compounds was screened. After identifying hit candidates through virtual screening, *in vitro* potency evaluation of hit candidates was performed to identify potent hits. The *in vitro* potency evaluation revealed 19 compounds with IC_50_ values in inhibiting Mpro below 100 μM, among them 2 compounds with very high potency with IC_50_ values below 1 µM.

## Results and Discussion

### Receptor-Rigid Docking on the NCI Library

**FIGURE 1:**
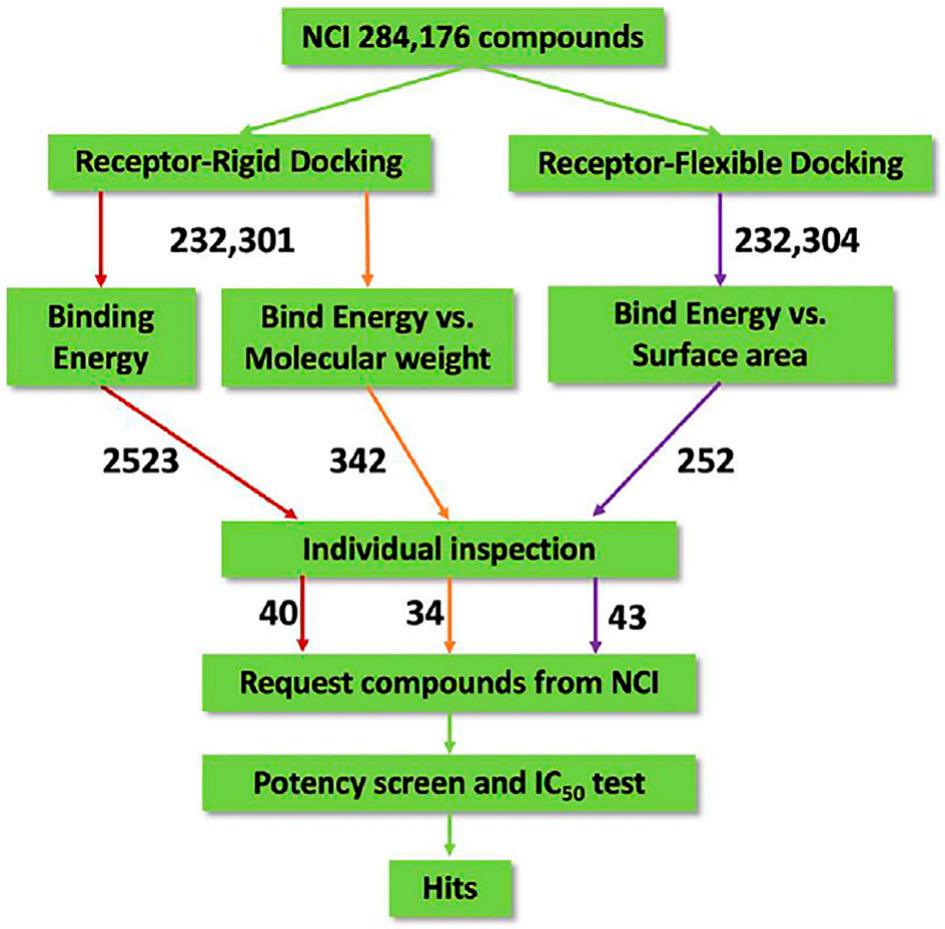
Flow diagram of the integrated virtual screen strategy. The diagram includes data preparation, docking methods, post-docking filtering, individual inspection, and *in vitro* potency test. The arrow is labeled with the number of compounds flowing along the respective colored path for assessment.

**FIGURE 2:**
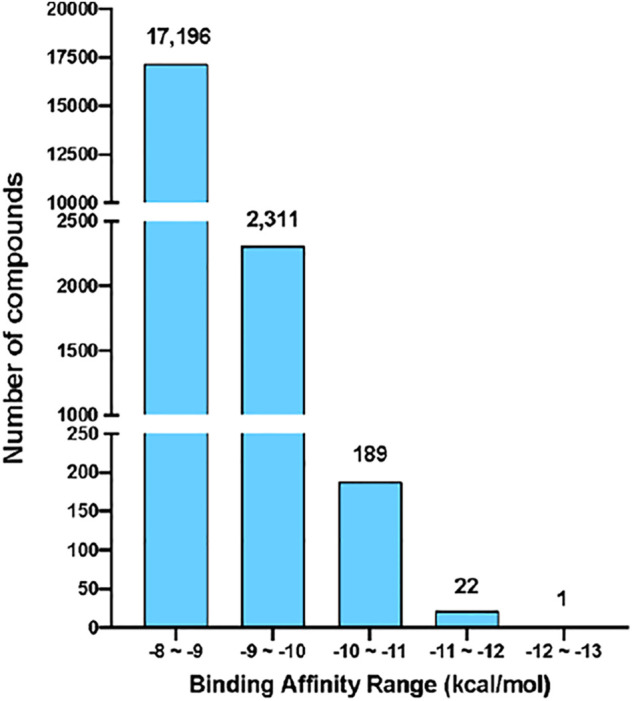
The binding energy distribution of receptor-rigid virtual screening results. Compounds with binding energy below −8 kcal/mol were categorized into five groups based on their binding energy values. The number of compounds in each group is shown on the top of the group bar.

The virtual screening process and strategy in this study are described in Figure 1 284,176 NCI compounds in the SDF format were downloaded and converted to the PDBQT format using the program OpenBabel. All prepared compounds were submitted to virtual screening against the active site of Mpro based on the PDB entry 6LU7 using the program Autodock Vina. Mpro has P1, P2 and P4 binding pockets for its substrates. These pockets were included during the docking process. After the receptor-rigid docking study, docking structures for 232,301 compounds were obtained. Each compound has at most 20 binding modes that were ranked based on their binding energy. Only the binding mode with the best binding energy of each compound was subsequently assessed. These docking structures were ranked based on their top binding energy, which led to 19,719 docking structures with top binding energy below −8 kcal/mol. The binding energy distribution of 19,719 compounds was calculated (Figure 2). The majority of compounds had binding energy between −8 kcal/mol to −9 kcal/mol. Only 2,523 compounds had binding energy lower than −9 kcal/mol. Compounds with binding energy lower than −9 kcal/mol were considered as promising hit candidates and further submitted for individual inspection. The individual inspection of compounds is based on three criteria: chemical correctness that assesses the 3D molecular conformations, pocket fitting that verifies that there are at least two fragments from a ligand fitted into two active site pockets of Mpro, and hydrophobicity that removes molecules that lead to favorable calculated binding energy apparently due to strong hydrophobicity of the compounds. Based on these criteria, we manually inspected the 2,523 receptor-rigid docking results with binding energy below −9.0 kcal/mol. 40 compounds were selected and requested from NCI for further *in vitro* potency testing.

Since binding energy correlates partially with the molecular weight, to further increase the accuracy of hit prediction, we also took the molecular weight of compounds into account. The distribution of binding energy *vs.* molecular weight was calculated (Table 1). 342 compounds with molecular weight between 200 and 400 Da and binding energy below −9 kcal/mol were considered as promising hit candidates and inspected individually. As a result of the inspection process, 34 out of 342 compounds were also requested from NCI for further *in vitro* potency testing. Please keep in mind that these 34 compounds were identified on the top of 40 compounds that were selected already by considering the contribution of the molecular size to the binding energy.

**TABLE 1:** Binding energy *vs*. molecular weight of virtual screening results.

Binding Affinity(kcal/mol)	−8 to −9	−9 to −10	−10 to −11	−11 to −12	−12 to −13
Molecular Weight(Da)					
0 to 100	—	—	—	—	—
100 to 200	—	—	—	—	—
200 t0 300	539	6	—	—	—
300 t0 400	5457	331	5	—	—
400 to 500	6502	786	40	2	—
500 t0 600	2811	648	37	6	—
600 t0 700	1038	294	40	3	—
700 t0 800	339	113	34	8	—
800 to 900	194	56	16	1	—
900 to 1000	110	20	7	1	—
1000 to 1200	49	14	5	1	1
1200 to 1300	37	10	1	—	—
1300 to 1400	30	14	—	—	—
1400 to 1500	16	7	—	—	—
1500 to 1600	5	4	—	—	—
1600 to 1700	2	1	1	—	—
1700 to 1800	5	—	—	—	—
1800 to 1900	4	1	—	—	—
1900 to 2000	1	—	—	—	—
2000 to 2100	2	—	—	—	—

### Receptor-Flexible Docking on the NCI Library

The virtual screening process in the receptor-flexible docking path was similar to that of the receptor-rigid situation, except that four residues were allowed to be flexible during docking. By inspecting potential interactions in the active site of Mpro involved in the binding of ligands, we defined H41, M49, N142 and Q189 as the four flexible residues. We obtained docking structures for 232,304 compounds and then ranked them based on their top binding energy. We analyzed 19,235 receptor-flexible docking results with molecular weights smaller than 400 Da and binding energy below −8 kcal/mol (Table 2). When compared to Receptor-Rigid docking, Receptor-Flexible docking resulted in more compounds with binding energy below -9 kcal/mol. This indicates that Receptor-Flexible docking may increase the chance to identify potential potent compounds for mimicking the solvent state of Mpro.

**TABLE 2:** Comparison of two binding energy distributions (receptor-flex *vs*. receptor-rigid) for compounds with molecular weight lower than 400 Da.

REeceptor/Energy Ranges	−8 to −9	−9 to −10	−10 to −11	−11 to−12	−12 to−13	Total
Receptor-Flexible	16,383	2,607	229	14	2	19,235
Receptor-Rigid	5996	337	5	—	—	6,338

**FIGURE 3:**
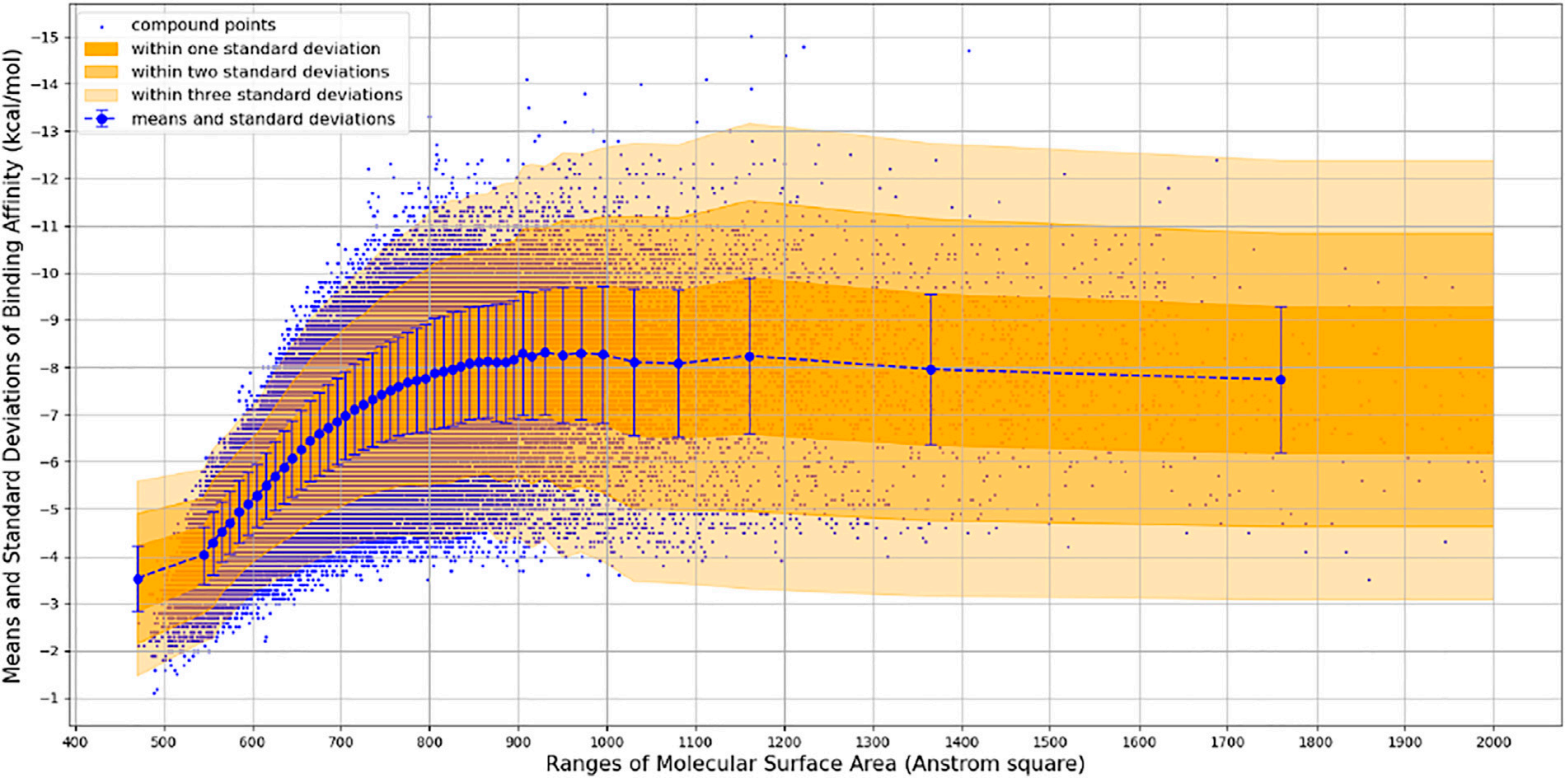
Binding Energy *vs*. Molecular Surface Area of Receptor-Flexible Docking Results. The surface areas are binned with 10 Å^2^, and then the mean and standard deviation of binding energy within each surface area bin is calculated. In order to get more reliable statistical results, we use a simple adaptive strategy to merge surface area bins so that each surface area bin contains at least 1,000 compound data points.

We observed that the number of promising hit candidates for further manual screening (2,852) is far more than that of receptor-rigid docking (342). In order to further narrow down the range of compound candidates, we further analyzed the distribution of binding energy *vs*. the molecular surface area (Figure 3). Here, we regard the molecular surface area as a more reliable metric than the molecular weight to measure the compound size. Most virtual screening programs calculate binding energy between a target and a ligand based on their potential van der Waals and other interactions that are heavily influenced by the compound size. The NCI library has a large compound library size that can be potentially statistically analyzed to reduce artificial influence on the binding energy by the compound size. By statistically analyzing binding energy *vs*. the molecular size, it can potentially allow us to obtain compounds with binding energy below the average level of each surface area group, and thus minimize potential artificial effects of the compound size in contributing to calculated binding energy. 252 compounds with molecular surface area smaller than 800 Å^2^ were selected with binding energy outside +3 standard deviations of the distribution of their corresponding surface area groups. We use 800 Å^2^ as a surface area cutting line since molecules with surface area bigger than 800 Å^2^ will be difficult for structure-activity relationship studies. Manual screening was also applied based on the chemical correctness, pocket fitting, and hydrophobic criteria outlined above. 43 compounds were selected and requested from NCI for further *in vitro* potency testing.

### Potency Screening and IC_50_ Determination for Selected Compounds From Receptor-Rigid Docking

**FIGURE 4:**
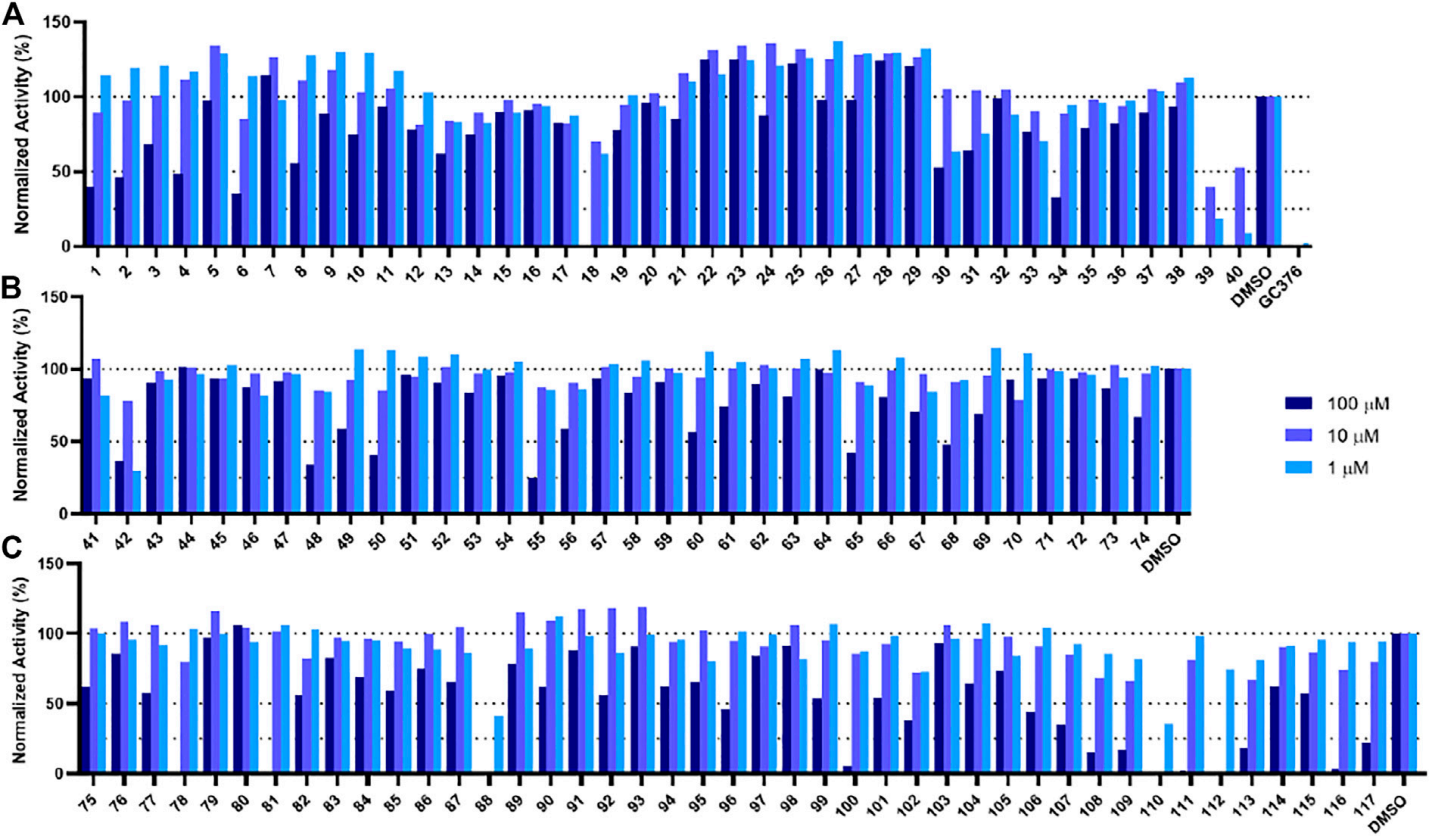
Initial screening of Mpro inhibition by selected compounds from docking. Tested compounds are selected from **(A)** the batch selected with affinity from receptor-rigid docking, **(B)** the batch selected with binding energy *vs*. molecular weight from receptor-rigid docking, and **(C)** the batch selected from receptor-flexible docking. 100, 10 and 1 µM were used for each inhibitor to perform the inhibition assay. Fluorescence intensity was monitored with respect to the control that had no inhibitor provided.

In order to test the potency of selected compounds from the rigid model-based molecular docking, two batches of a total of 74 compounds were requested from NCI. The first batch contained 40 compounds which were selected based on just binding energy (below -9 kcal/mol), and the second batch contained 34 compounds which were selected based on the relationship between binding energy and molecular weight. These compounds were dissolved in DMSO to a concentration of 10 mM and stored at −20°C for further usage. The potency of selected compounds was screened at a concentration of 100 μM, 10 and 1 µM. First, the inhibitors were incubated with 50 nM Mpro at 37°C for 30 min. Then the reaction was initiated by adding 100 µM of a fluorescent substrate Sub3 (Vatansever et al., 2021a). The assay was monitored by a plate reader with Ex336/Em455 for 30 min. The first 10 min was fitted with linear regression by GraphPad Prism. The initial slope value was used as normalized activity. GC376 was tested as a control (Yang et al., 2021). As shown in Figure 4A, at 100 μM, 8 out of 40 compounds displayed more than 50% inhibition of the activity of Mpro (Compounds 1, 2, 4, 6, 18, 34, 39, and 40). These 8 compounds were subjected to a more thorough IC_50_ assay (Supplementary Figure S2). Five compounds had a determined IC_50_ value below 100 µM. Notably, compounds 39 and 40 show potent inhibition with IC_50_ values as 17.9 and 19.4 µM, respectively (Supplementary Figure S5, Supplementary Table S1). In the batch with the molecular weight considered (Figure 4B), 6 out of 34 compounds exhibited more than 50% inhibition of Mpro activity (Compounds 42, 48, 50, 55, 65, 68). 4 out of these 6 compounds had an IC_50_ value below 100 µM. If we consider the hits rate as IC_50_ less than 50 μM, the average hit rate for rigid-docking is about 2.7%. All the tested compound structures can be found in Supplementary Figure S1, and docking poses of potent compounds are shown in Supplementary Figure S3. Our results clearly showed that by taking the contribution of the molecular size to the calculated binding energy into account, additional compounds with high potency can be identified.

### Potency Screening and IC_50_ Determination for Selected Compounds From Receptor-Flexible Docking

**FIGURE 5:**
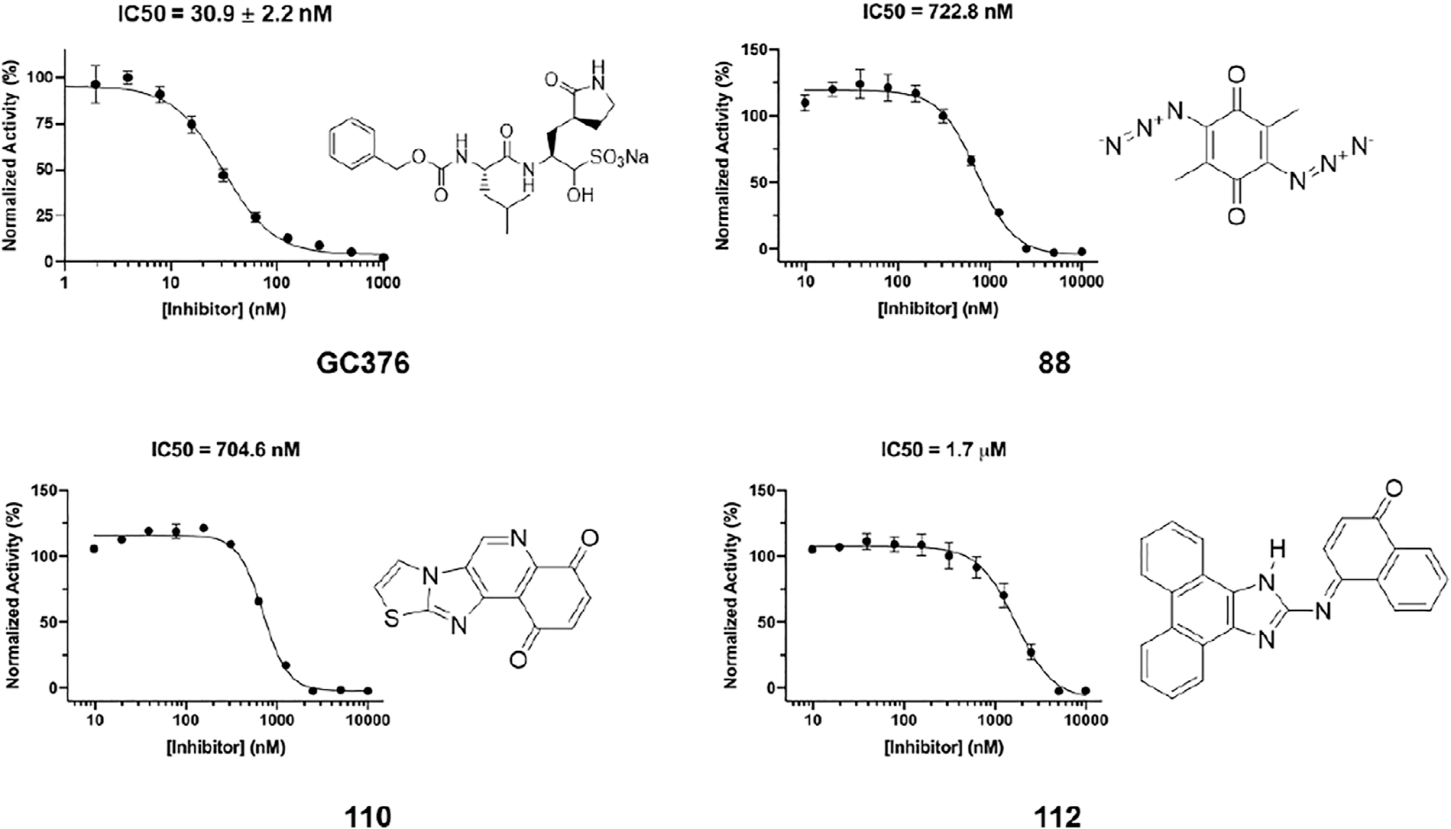
IC_50_ determination of selected compounds from the docking process against Mpro. GC376 was tested as control. Triplicate experiments were performed for each compound. GraphPad Prism 8.0 was used to perform data analysis.

In order to test the potency of selected compounds from flexible model-based molecular docking, 43 selected compounds were requested from NCI. The same protocol as the rigid-model docking batch was applied. The initial 100 µM screening shows 16 compounds out of 43 suppressed the activity of Mpro by more than 50% (Figure 4C). Those compounds which inhibited activity more than 75% were subjected to an IC_50_ test (Supplementary Figure S2). Eleven compounds out of 12 had an IC_50_ of less than 100 μM, 7 compounds had an IC_50_ value less than 50 µM. The hit rate for this batch is 16% (7/43), which is dramatically higher than the rigid-model docking result. This result indicates that the flexible docking method significantly increases the hit candidate rate and lowers the percentage of false positives. Compounds 78, 88, 109, 110, 111 and 112 showed potent IC_50_ values of 13.3, 0.723, 12.8, 0.705, 10.3 and 1.69 µM respectively (Figure 5, Supplementary Figure S2, Supplementary Table S1). These 6 compounds exhibit critical and competitive potency *in vitro* when compared to current non-covalent Mpro inhibitors. Among them, compounds 88, 110 and 112 could covalently react with the active site C145 of Mpro. Considering these three compounds are all quinones that could oxidize the catalytic cysteine of Mpro instead of binding, we test all of them on another cysteine protease of SARS-CoV-2, PLpro. The results should show that these three compounds are more selective on Mpro than PLpro, and confirm that at least compound 88 and 110 specifically inhibit the activity of Mpro (Supplementary Figure S4). All the tested compound structures can be found in Supplementary Figure S1, and docking poses of potent compounds are shown in Supplementary Figure S3. Our results clearly demonstrated that the combination of flexible model-based docking and the statistical analysis of binding energy *vs*. molecular size to identify molecules with high binding energy deviation from the average binding energy of its belonged group is an optimal approach to narrow down compound candidates with high potency.

## Conclusion

Since 2003, there have been three coronavirus disease outbreaks. Researchers have predicted that additional coronavirus diseases will emerge with higher frequency. For both combating the current pandemic and preparing to contain future coronavirus disease outbreaks, it is imperative to develop small molecule antivirals that can be applied generally to inhibit coronaviruses. Due to its conserveness among coronaviruses, Mpro is an attractive drug target for broad-spectrum antivirals. In this study, we performed both Receptor-Rigid docking and Receptor-Flexible docking on the NCI compound collection that contains 284,176 compounds. The docking results were further processed using a refined strategy. The binding energy *vs*. molecular weight filter was applied to the receptor-rigid docking results, and the binding affinity *vs*. surface area filter was applied to the receptor-flexible docking results. After docking, individual inspection was conducted based on the chemical correctness, pocket fitting and hydrophobic criteria. We show that this strategy has significantly increased the accuracy rate compared to the standard virtual screening method, which only ranks by binding energy. The feasibility of our approach has been validated by the *in vitro* potency testing results, which led to the identification of several potent inhibitors. Two inhibitors have IC_50_ values below 1 μM, making them among most potent Mpro inhibitors that have been discovered so far. Almost all inhibitors that have been discovered in this study are reported for the first time. Their mechanisms of action need to be explored for aiding structure-activity relationship studies to identify more potent inhibitors with drug-like features as preclinical candidates for COVID-19.

Although the enhanced hybrid screening approach has been successfully developed and applied in searching for potent drug candidates, there are still some challenges that need to be conquered as well as improvement that could be explored in the following research. First, the outcome of receptor-flexible docking relies on the residues chosen. The four residues we choose in this paper are based on our previous crystallographic structure study of Mpro (Yang et al., 2021). Better understanding of the interactions between target and inhibitors will be critical in making the choices of residues for flexible docking. Second, the individual inspection dramatically relied on personal experience and knowledge. Finding a method to precisely evaluate the potential potency of inhibitors instead of using manual inspection will be a good future exploration. Third, *in vitro* screens and IC_50_ tests for large amounts of potential compounds are a time- and labor-consuming step. A more efficient way, such as incorporation of high throughput screening, would significantly accelerate the *in vitro* potency test.

## Materials and Methods

### Chemicals

All compounds used in this study were requested from The Developmental Therapeutics Program (DTP) of National Cancer Institute (NCI) without further purification and characterization (Monga and Sausville, 2002).

### Protein Preparation of SARS-CoV-2 Mpro

The crystal structure of the SARS-CoV-2 Mpro in complex with an N3 inhibitor (PDB ID: 6LU7) was obtained from the RCSB Protein Data Bank (https://www.rcsb.org/). Only chain A of the structure was used in our docking-based virtual screening studies. The cognate ligand was extracted from the structure. In AutoDockTools-1.5.7 (Sanner, 1999; Morris et al., 2009), water molecules were deleted, and polar hydrogens were added to the structure. Finally, the prepared protein structure was converted into a PDBQT file for further receptor-rigid docking studies.

In addition to receptor-rigid docking, we also conducted receptor-flexible docking, which was motivated by our two observations. One is that residues Met49 and Asn142 were observed to cause significant conformation changes at the active site. The other one is that residues His41 and Gln189 were observed to form important interactions with inhibitors (Yang et al., 2021). Considering these structural characteristics, we made side chains of residues Met49, Asn142, His41 and Gln189 flexible. Similarly, we used AutoDockTools-1.5.7 (Sanner, 1999; Morris et al., 2009) to delete water molecules, add polar hydrogens, and then split the receptor into a rigid part and a flexible part. Both of the prepared rigid part and the flexible part of the protein structure were converted into PDBQT files for further receptor-flexible docking studies.

### Ligand Preparation of NCI Open Chemicals Repository

The DTP of NCI maintains a repository with synthetic compounds and pure natural products that are available at no cost to investigators for non-clinical research purposes (Monga and Sausville, 2002). The repository collection is a uniquely diverse set of more than 200,000 compounds. A collection of 284,176 2D compound structures in SDF format are also provided. We converted these 2D SDF files into 3D PDBQT files using OpenBabel-3.1.1 (O'Boyle et al., 2011) with the “--gen3d dg” option. A total of 279, 442 compounds were successfully converted.

### Docking Parameters and Method

In addition to receptor and ligand preparations, AutoDockTools-1.5.7 (Sanner, 1999; Morris et al., 2009) was also used for grid parameter setting. The cognate ligand of crystal structure 6LU7 suggested the inhibitor binding site. A grid box with dimensions 30 × 30 × 30 centered at the coordinates X = − 10.0, Y = 13.0, and Z = 70.0 was used to represent the search space. Then we applied AutoDock Vina (Trott et al., 2010) docking protocol with options of 8 CPUs to use and maximum 20 binding modes to generate. Only the top binding energy and binding modes were shown in this paper. In order to speed up the virtual screening process, the commands for both compound format conversion and molecular docking of 279, 442 compounds were distributed among more than 6, 000 requested CPUs from Texas A&M High Performance Research Computing Clusters.

### Recombinant Mpro Protein Expression and Purification

The pET28a-His-SUMO-Mpro construct was made based on a pET28a plasmid modified with an N-terminal His-SUMO tag. The gene encoding Mpro was amplified from a previous plasmid pBAD-sfGFP-Mpro using the forward primer 5′-CGC​GGA​TCC​GGG​TTT​CGC​AAG-3′ and the reverse primer 5′- CCG​CTC​GAG​TTA​CTG​AAA​AGT​TAC​GCC-3′. The amplified PCR product was digested by *BamHI* and *XhoI* and ligated into the vector pET28a-His-SUMO plasmid that was digested with the same restriction enzymes. The gene sequence of His-SUMO-Mpro was verified by sequencing at Eton Bioscience Inc.

The pET28a-His-SUMO-Mpro construct was transformed into *E. coli* BL21 (DE3) cells. Transformed cells were cultured at 37°C in 6 L 2xYT medium with kanamycin (50 g/ml) for 3 h and induced with isopropyl-D-1-thiogalactoside (IPTG) at final concentration of 1 mM when the OD_600_ reached 0.8. After 3 h, cells were harvested by centrifugation at 12,000 rpm, 4°C for 30 min. Cell pellets were resuspended in 150 ml buffer A (20 mM Tris, 100 mM NaCl, 10 mM imidazole, pH 8.0) and then lysed by sonication on ice. The lysate was clarified by centrifugation at 16,000 rpm, 4°C for 30 min. The supernatant was loaded onto a nickel-chelating column with high affinity Ni-charged resin from GenScript and washed with 10 column volumes of buffer A to remove unspecifically bound proteins, which was followed by elution using buffer B (20 mM Tris, 100 mM NaCl, 250 mM imidazole, pH 8.0). The protein eluates were subjected to buffer exchange with buffer C (20 mM Tris, 10 mM NaCl, 1 mM dithiothreitol (DTT), pH 8.0) by using a HiPrep 26/10 desalting column (GE Healthcare). The His-SUMO-Mpro proteins were digested with SUMO protease overnight at 4°C. The digested protein was applied to a nickel-chelating column again to remove the His-tagged SUMO protease, the His-SUMO tag, and the expressed protein with uncleaved His-SUMO tag. The tag-free Mpro protein was loaded onto an anion-exchange column with Q Sepharose, Fast Flow (GE Healthcare) equilibrated with buffer C for further purification. The column was eluted by buffer D (20 mM Tris, 1 M NaCl, 1 mM DTT, pH 8.0) with a linear gradient ranging from 0 to 500 mM NaCl. Fractions eluted from the anion exchange column were condensed and loaded to a size exclusion column with HiPrep 16/60 Sephacryl S-100 HR (GE Healthcare) pre-equilibrated with buffer E (20 mM Tris, 100 mM NaCl, 1 mM DTT, 1 mM EDTA, pH 7.8). The eluted Mpro protein in buffer E was concentrated to 20 mg/ml and stored in −80°C for further use.

### IC_50_ Analysis

The assays were carried out with 50 nM enzyme and 10 µM substrate at 37 °C with continuous shaking. The Sub3 substrate (DABCYL-Lys-Thr-Ser-Ala-Val-Leu-Gln-Ser-Gly-Phe-Arg-Lys-Met-Glu-EDANS) was purchased from BACHEM and stored as 1 mM solution in 100% DMSO. Enzyme activity was monitored by detecting fluorescence with excitation at 336 nm and emission at 455 nm wavelength. The dilution buffer (used for enzyme and substrate dilution) is 10 mM Na_x_H_y_PO_4_, 10 mM NaCl, 0.5 mM EDTA, pH 7.6. Final composition of the assay buffer is 10 mM Na_x_H_y_PO_4_, 10 mM NaCl, 0.5 mM EDTA, 2 µM DTT (coming from enzyme stock solution), pH 7.6 with 1.25% DMSO. All inhibitors were stored as 10 mM in 100% DMSO solutions in a −20°C freezer.

For the IC_50_ analysis, the inhibitor was diluted to 400-fold times higher than the highest working concentration to make the secondary stock solution (i.e., if the highest working concentration of inhibitor is 2 μM, then the inhibitor was diluted from its 10 mM stock solution to 800 µM in DMSO). 10 µL from this secondary stock solution was added to 990 uL of the dilution buffer. Serial dilutions were carried out in the dilution buffer containing 1% DMSO to ensure all the inhibitor serial dilutions contained 1% DMSO. 25 µL of each inhibitor solution were added to a 96-well plate with a multichannel pipettor. Next, 25 µL of a 200 nM enzyme solution (diluted from 10 µM enzyme storage solution in 10 mM Na_x_H_y_PO_4_, 10 mM NaCl, 0.5 mM EDTA, pH 7.6, 1 mM DTT in the dilution buffer) was added by a multichannel pipettor and mixed by pipetting up and down three times. Then, the enzyme-inhibitor solution was incubated at 37°C for 30 min. During the incubation period, 20 µM of the substrate solution was prepared by diluting from 1 mM stock solution in the dilution buffer. When the incubation period was over, 50 µL of the 20 µM substrate solution was added to each well using a multichannel pipettor and the assay started. Data recording was stopped after 30 min. Data treatment was done with GraphPad Prism 8.0. The first 0–300 s were analyzed by linear regression for initial slope analyses. Then, the initial slopes were normalized and IC_50_ values were determined by inhibitor *vs* response - Variable slope (four parameters).

## Data Availability

The raw data supporting the conclusions of this article will be made available by the authors, without undue reservation.
