# Supplementary material for: An Enhanced Hybrid Screening Approach to Identify Potent Inhibitors for the SARS-CoV-2 Main Protease From the NCI Compound Library
Source: Front Chem. 2022 Feb 17;10:816576. doi: 10.3389/fchem.2022.816576 (PMC8892251; doi:10.3389/fchem.2022.816576)
Supplement: Supplementary file 1 [file DataSheet1.PDF]

Supplementary Information for

An Enhanced Hybrid Screening Approach to Identify Potent Inhibitors for the SARS-CoV-2 Main Protease from the NCI Compound Library

Shuhua Gianna Li[a,†], Kai S. Yang[a,†], Lauren Blankenship[a], Chia-Chuan Dean Cho[a], Shiqing Xu[a,*], Hongbin Wang[b,*], Wenshe R. Liu[a,c,d,e,*]

[a]Texas A&M Drug Discovery Laboratory, Department of Chemistry, Texas A&M University, College Station, TX 77843, USA

[b]Center for Biomedical Informatics, Texas A&M University Health Science Center, Houston, TX 77030; USA

[c]Institute of Biosciences and Technology and Department of Translational Medical Sciences, College of Medicine, Texas A&M University, Houston, TX 77030, USA

[d]Department of Biochemistry and Biophysics, Texas A&M University, College Station, TX 77843, USA

[e]Department of Molecular and Cellular Medicine, College of Medicine, Texas A&M University, College Station, TX 77843, USA

[†]Contributed equally to the paper.

*Correspondence should be addressed to Shiqing Xu: shiqing.xu@tamu.edu, Hongbin Wang: hwang@tamhsc.edu and Wenshe Ray Liu: wsliu2007@tamu.edu

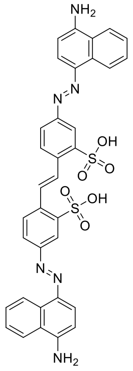
1 (nsc 5067)

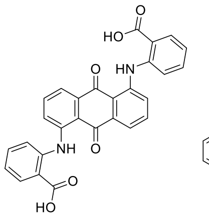
2 (nsc 16221)

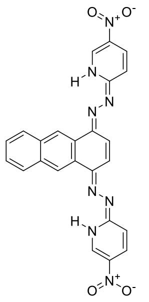
4 (nsc 43996)

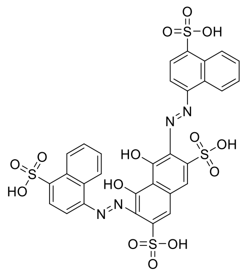
6 (nsc 74512)

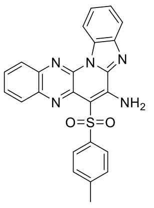
18 (nsc 648405)

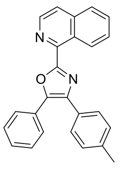
39 (nsc 116702)

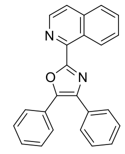
40 (nsc 116704)

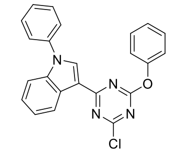
42 (nsc 142335)

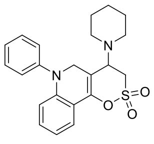
48 (nsc 312335)

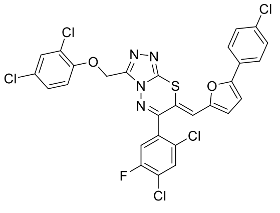
34 (nsc 719147)

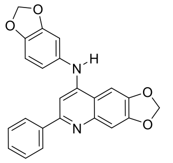
50 (nsc 346551)

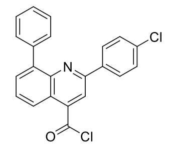
55 (nsc 400924)

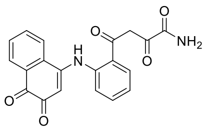
65 (nsc 641243)

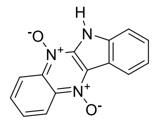
78 (nsc_034561)

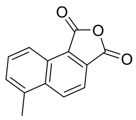
81 (nsc_062795)

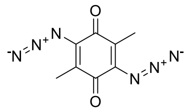
88 (nsc_110653)

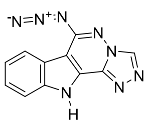
100 (nsc_360522)

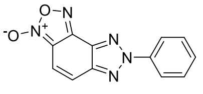
108 (nsc_663966)

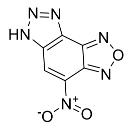
109 (nsc_669452)

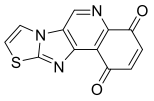
110 (nsc_725107)

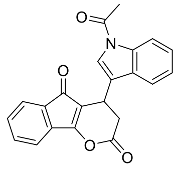
111 (nsc_208736)

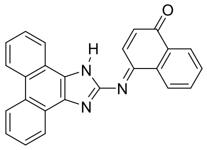
112 (nsc_342738)

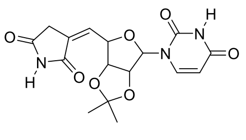
113 (nsc_344288)

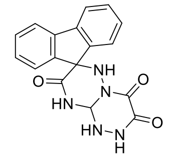
116 (nsc_666169)

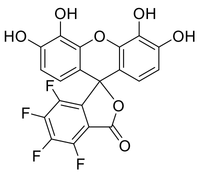
117 (nsc_726285)

Figure S1. Structure of compounds with measurable potency.

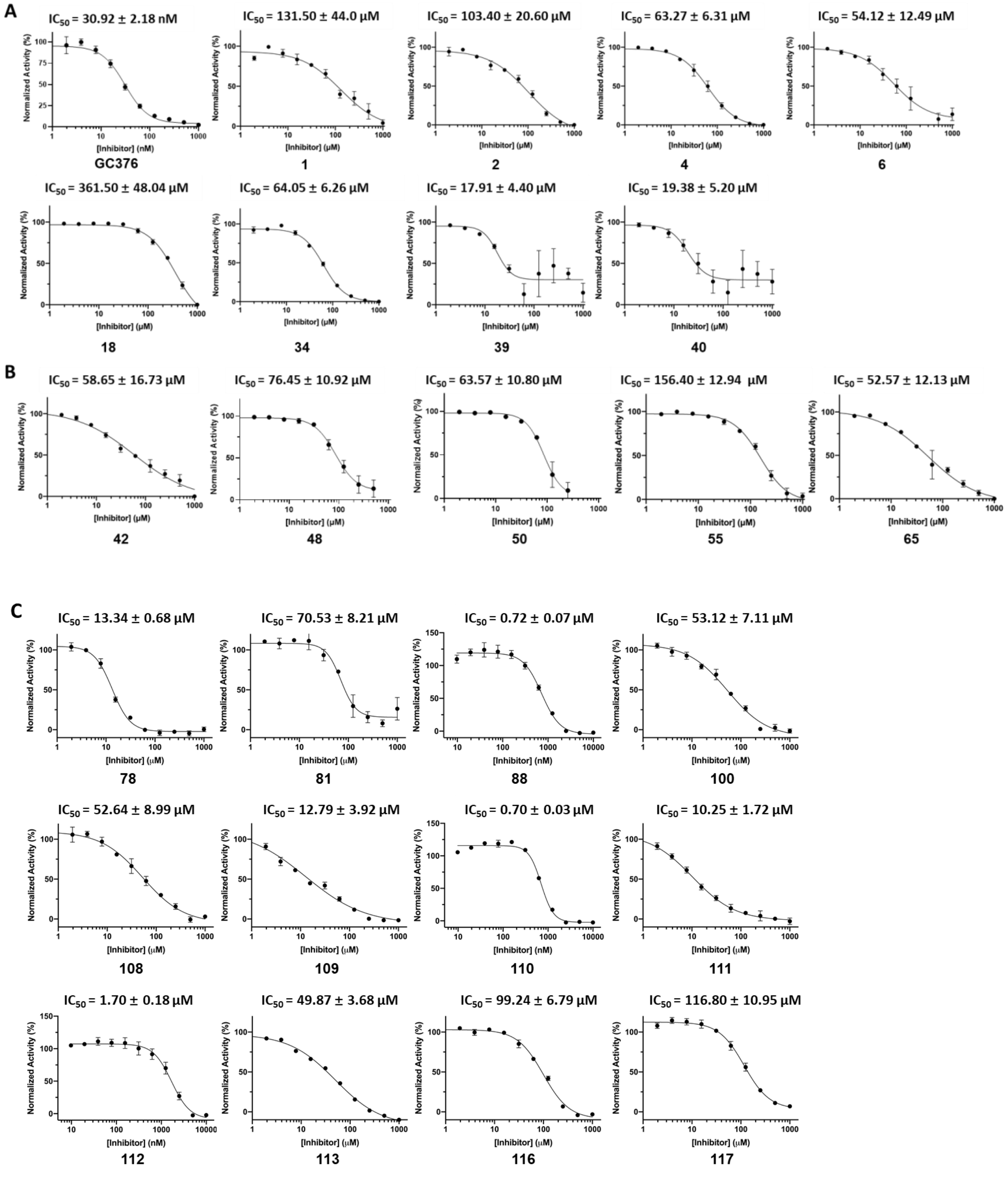

Figure S2. IC_{50} determination of selected compounds. Triplicate experiments were performed for each compound. GraphPad Prism 8.0 was used to perform data analysis.

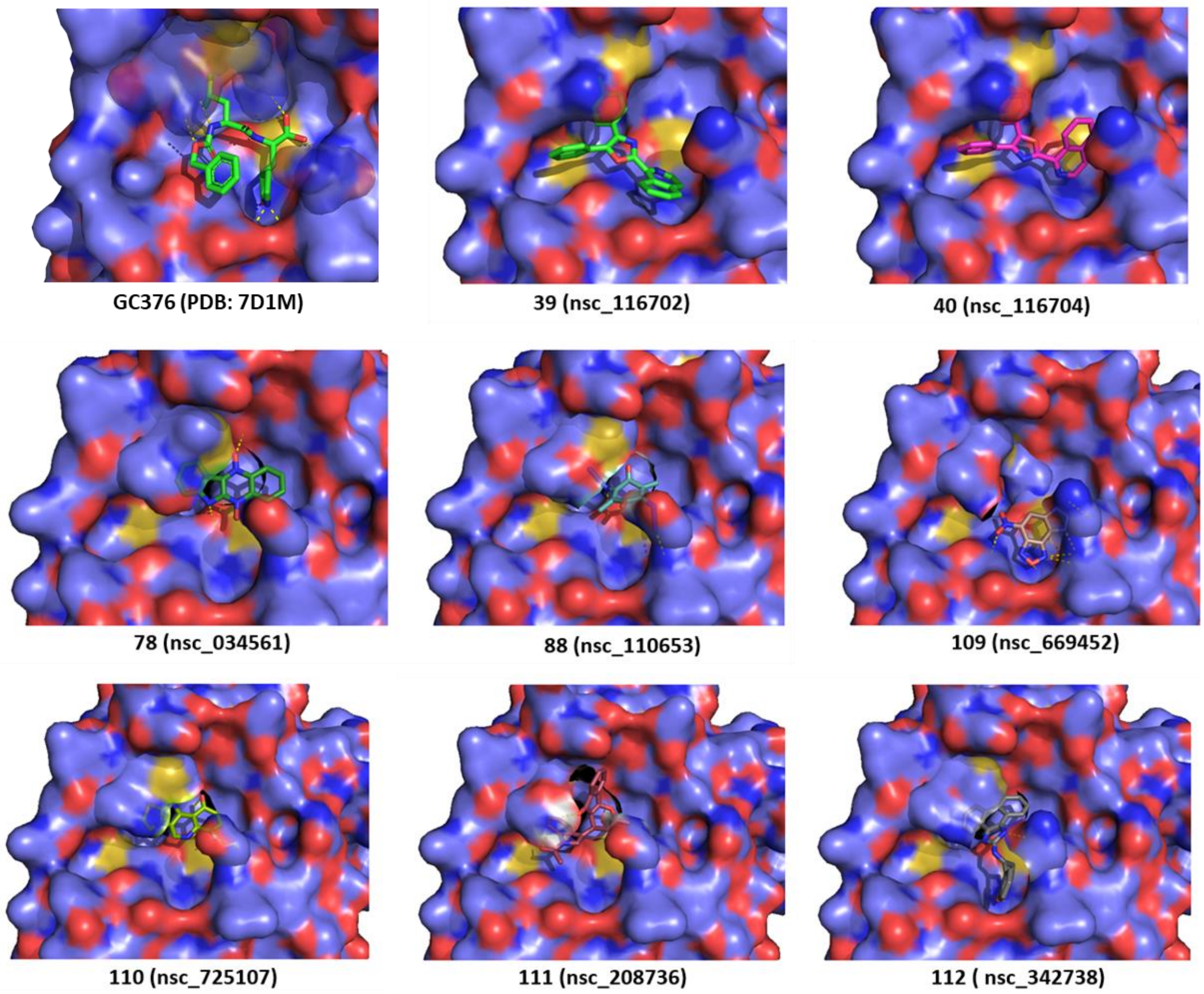

Figure S3. Docking pose of selected compounds. Crystal structure of Mpro with GC376 is presented as a comparison (PDB ID 7D1M) [1]. All structural figures were generated with PyMOL (https://www.pymol.org).

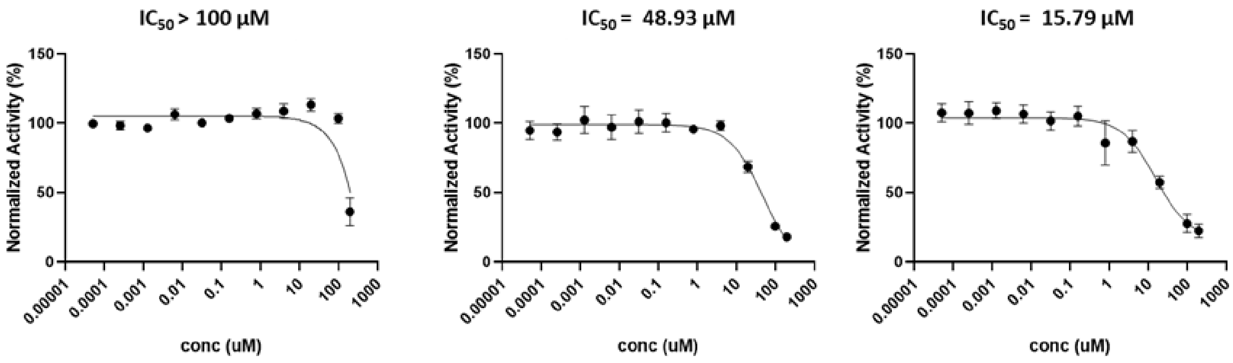

88 **110** **112**

Figure S4. IC_{50} of compound 88, 110 and 112 against PLpro. The IC_{50} test methods are available in the previous report [2].

Table S1. Binding energy and IC_{50} values of compounds with measurable potency.

NCI Compound ID	Binding Energy (kcal/mol)	IC_{50} (μM)	NCI Compound ID	Binding Energy (kcal/mol)	IC_{50} (μM)
**1 (nsc_5067)**	-9.8	132 ± 44	**78 (nsc_034561)**	-8.7	13.3 ± 0.7
**2 (nsc_16221)**	-9.8	103 ± 21	**81 (nsc_062795)**	-8.3	70.5 ± 8.2
**4 (nsc_43996)**	-10.1	63.3 ± 6.3	**88 (nsc_110653)**	-7.6	0.723 ± 0.066
**6 (nsc_74512)**	-9.8	54.1 ± 12.5	**100 (nsc_360522)**	-8.3	53.1 ± 7.1
**18 (nsc_648405)**	-10.2	362 ± 48	**108 (nsc_663966)**	-8.8	52.6 ± 9.0
**34 (nsc_719147)**	-9.5	64.1 ± 6.3	**109 (nsc_669452)**	-7.2	12.8 ± 3.9
**39 (nsc_116702)**	-9.4	17.9 ± 4.4	**110 (nsc_725107)**	-8.3	0.705 ± 0.034
**40 (nsc_116704)**	-9.1	19.4 ± 5.2	**111 (nsc_208736)**	-10.3	10.3 ± 1.7
**42 (nsc_142335)**	-9.2	58.7 ± 16.7	**112 (nsc_342738)**	-10.5	1.69 ± 0.18
**48 (nsc_312335)**	-9.3	76.5 ± 10.9	**113 (nsc_344288)**	-10.7	49.9 ± 3.7
**50 (nsc_346551)**	-9.1	63.6 ± 10.8	**116 (nsc_666169)**	-10.1	99.2 ± 6.8
**55 (nsc_400924)**	-9.0	156 ± 13	**117 (nsc_726285)**	-11.1	117 ± 11
**65 (nsc_641243)**	-9.0	52.6 ± 12.1			

Reference

1. Fu, Lifeng et al. “Both Boceprevir and GC376 efficaciously inhibit SARS-CoV-2 by targeting its main protease.” Nature communications vol. 11,1 4417. 4 Sep. 2020, doi:10.1038/s41467-020-18233-x

2. Cho, Chia-Chuan et al. “Drug Repurposing for the SARS-CoV-2 Papain-Like Protease.” ChemMedChem vol. 17,1 (2022): e202100455. doi:10.1002/cmdc.202100455
